# Analysis of conventional and alternative CRISPR/Cas9 genome editing to enhance a single-base pair knock-in mutation

**DOI:** 10.1186/s12896-021-00707-5

**Published:** 2021-07-27

**Authors:** Carina Edmondson, Qi Zhou, Xuan Liu

**Affiliations:** Department of Biochemistry, University of California, Riverside, CA 92521 USA

**Keywords:** CRISPR/Cas9, Knock-in mutation, Base editing efficiency

## Abstract

**Background:**

The use of CRISPR/Cas9 technologies in generating single-base pair knock-in mutations has recently exploded in the number of methods available. However, with the growing expansion of new technologies, it can be difficult to determine the best method for genome editing.

**Results:**

In this study, we evaluated a number of CRISPR/Cas9 approaches for deriving cell lines with knock-in base pair edits to create a phosphorylation mutation and provide a breakdown of editing efficiencies and suggestions for improvement. Overall, our studies suggest that using pre-formed ribonucleoprotein (RNP) complexes is a reliable editing method to generate homozygous single-base pair mutations. We also show that antibiotic selection coupled homologous recombination is an efficient tool for generating highly specific heterozygous mutations.

**Conclusion:**

The methods and/or combination of methods outlined in this study can be used to help other researchers with similar goals in single-base pair genome editing.

**Supplementary Information:**

The online version contains supplementary material available at 10.1186/s12896-021-00707-5.

## Introduction

Cell signaling is a crucial method of communication that controls the ability of cells to respond to environmental cues, resulting in the coordination of multiple cascades important for growth and survival. A main regulatory feature of the signal transduction pathways responsible for this response is protein phosphorylation, a reversible post-translational modification of serine/threonine (S/T), or tyrosine residues (Y) [1]. Because phosphorylation is a key regulator of signaling pathways, studying the mechanisms of phosphorylation and how specific phosphorylation sites contribute to downstream cellular events is crucial to our understanding of how a cell works [2]. Many studies of protein phosphorylation include small chemical inhibitors, or RNAi knockdowns of protein kinases or phosphatases [3]. More recently, genomic editing of key players involved in signaling pathways allows researchers to knock out genes or knock-in mutations of a gene to study function of phosphorylation.

Early technologies of genome editing, including recombinant adeno-associated viruses (rAAV), meganucleases, zinc-finger nucleases (ZFNs), and transcription activator-like effector nucleases (TALENs), had major limitations in their capability to provide a cost effective, highly efficient, and adaptable technology [4–8]. After the introduction and discovery of the innate immune response of bacteria, the CRISPR/Cas9 system became a revolutionary genome editing tool [9]. The CRISPR, or Clustered Regularly InterSpaced Palindromic Repeats, locus from the type II *Streptococcus pyogenes* (*Sp*) has been utilized for editing due to its characteristic DNA targeting and cleaving capabilities. This locus contains cas (CRISPR-associated) genes including Cas9 nuclease, a trans-activating CRISPR RNA (tracrRNA), and CRISPR RNA (crRNA) containing direct repeats interspaced with variable protospacers [10]. By using a two-component CRISPR/Cas system encompassing *Streptococcus pyogenes* Cas9 (*Sp*Cas9) and chimeric crRNA-tracrRNA hybrid (gRNA), targeted double stranded breaks (DSBs) can be generated in mammalian cells [11, 12].

Following DNA DSBs, either the Non-homologous End Joining (NHEJ) or Homology-Directed Repair (HDR) pathway is activated [13, 14]. NHEJ results in insertion or deletion (indel) mutations that result in frameshift mutations, leading to loss of protein function in the target gene (knock-out). In contrast, HDR results in specific mutations such as single base pair modifications, or large insertions (knock-in) due to the presence of a donor template. To enhance the precision of single base pair edits, a new modification to the CRISPR/Cas9 system allows for targeted single base pair editing from A:T to G:C. The technology utilizes an adenosine deaminase fused to a catalytically impaired Cas9 (dCas9), thus no DSBs are created, eliminating the need for a DONOR template and therefore enhancing editing efficiencies exclusive of indel mutations [15]. Through the precision of CRISPR/Cas9-mediated genome editing, stable cell lines can be produced to harbor specific mutations and allow study of the effect of the mutation on molecular pathways.

**Fig. 1:**
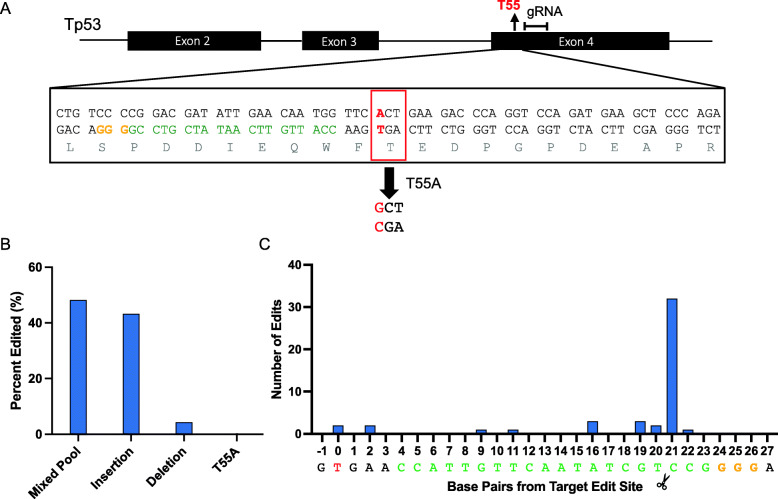
Traditional plasmid-based CRISPR/Cas9 genome editing results in high incidence of indel mutations. **A** Design of gRNA (green) and PAM sequence (orange) in respect to location of T55 residue (red box). **B**, **C** Percentage of specific mutations (**B**) and their location (**C**) from the target edit site (designated as 0}. Red indicates target edit and scissors indicate Cas9 cut site. Note data represents sequence data of template strand

Tumor suppressor p53, commonly known as the guardian of the genome, is well-known to be regulated by post translational modification, including phosphorylation [16]. Recently, we have discovered a new mechanism for the regulation of p53 by TAF1 kinase, the largest subunit of transcription factor II D (TFIID) [17]. Phosphorylation of p53 by TAF1 kinase at Thr55 results in the dissociation of p53 and TAF1 from the p21 promoter, leading to transcription inactivation and subsequent p53 protein degradation [18]. To determine the function of Thr55 phosphorylation, we aimed to mutate this residue from threonine (T) to alanine (A) (Fig. 1A) to create a knock-in T55A cell line for further analysis. However, CRISPR/Cas9 knock-in editing relies on HDR (compared to creation of a knock-out edit with NHEJ) that accounts for a much lower efficiency of DNA repair, thus making knock-ins and single base pair edits more difficult. To circumvent this problem, a number of methods to either enhance knock-in editing by inhibiting NHEJ or by avoiding DSBs have been established, aiming to reduce incidence of indel mutations. Here we compare and review the efficacy of those CRISPR/Cas9 based methods to create a knock-in T55A cell line .

## Results

### Traditional plasmid-based CRISPR/Cas9 genome editing results in high incidence of indel mutations

**Fig. 2:**
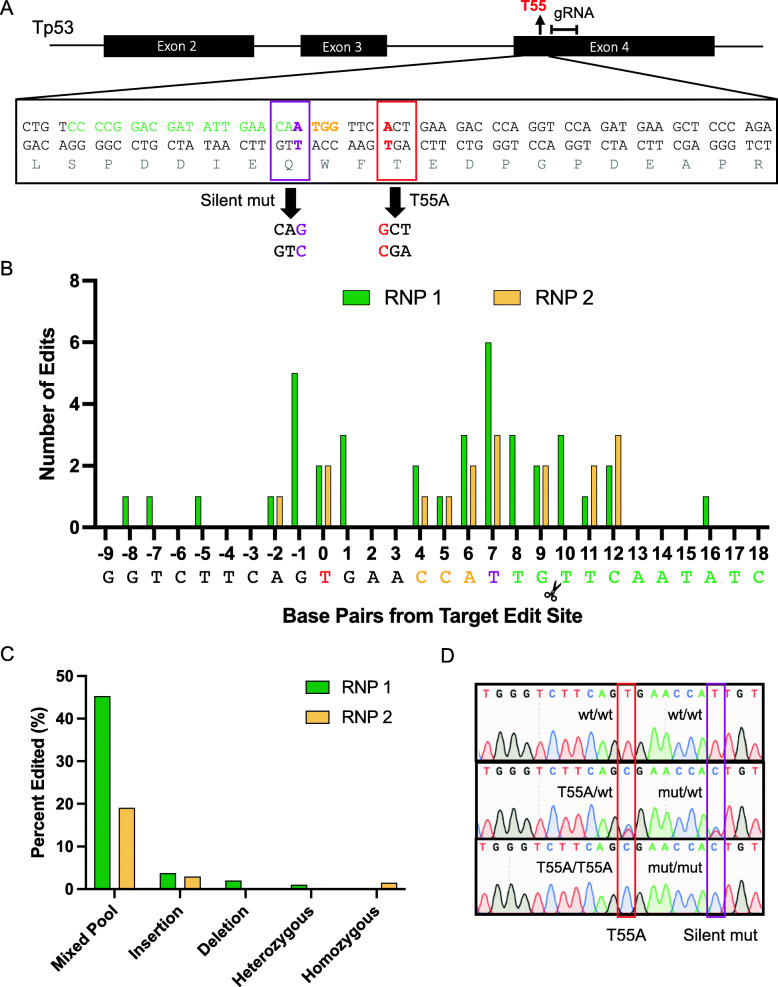
Ribonucleoprotein (RNP) complex, the proximity of the cut to the edit site, and inclusion of a silent mutation increase efficiency of single base pair edits. **A** Design of gRNA (green), PAM (orange), silent mutation (purple box) in respect to target T55 location (red box). **B**, **C** Locations and types of mutations in the first round of RNP (RNP 1, green) and the second round of RNP (RNP 2, yellow) of editing. Scissors indicate Cas9 cut site. **D** Chromatogram of Wildtype (top), heterozygous (middle), and homozygous (bottom) T55A mutations. Note data represents sequence data of template strand

Traditional methods of CRISPR/Cas9 rely on transfection of a plasmid that co-expresses the Cas9 protein and gRNA [11, 12]. Upon transcription and translation (if applicable), the gRNA and Cas9 nuclease undergo complex formation to create the Cas9:gRNA complex (Figure S1). Multiple features of the gRNA contribute to the efficiency in which editing can occur, including alignment to a PAM sequence, proximity of the guide to the target edit site, and consideration of potential off-target effects. To maximize efficiency of a single base pair edit, two best options for gRNA design (with Quality Score 97 and 91, Table S1) were selected so that the cut site was within 20 base pairs from the target edit, limited by the presence of the “NGG” PAM sequence and potential off target effects (Figs. 1A and 2A). The gRNA with less off target sites (#1 gRNA, Table S1 and Fig. 1A) was cloned into the pSpCas9(BB)-2A-Puro plasmid and transfected into a mammalian cell line (HCT116) along with a single-stranded oligodeoxynucleotide (ssODN) DONOR template harboring T55A mutation (Table S2). In addition, SCR7, a NHEJ inhibitor, was used to increase HDR occurrence [19]. Following puromycin selection, DNA was sequenced from 49 individual clones (Table S3 and S4). As shown in Fig. 1B, the majority of clones contains “mixed pool” or insertion mutations, caused by indels. Note that “mixed pool mutations” refer to a mixture of heterozygous indel mutations that are created likely due to imperfect HDR repair following nuclease activity [20]. Furthermore, our analysis shows that edits occurred mainly within the gRNA sequence, between the target and the Cas9 cut site, with the highest mutation rate at the cut site (Fig. 1C). The large incidence of indel mutations suggests that NHEJ occurred frequently despite the presence of SCR7. Overall, while the traditional plasmid-based method generates 96% editing (Fig. 4A), due to high occurrence of NHEJ, the resulting mutations did not produce the desired T55A knock-in mutation.

### Ribonucleoprotein (RNP) complex coupled with a silent mutation and proximity of the edit site increase efficiency of single base pair edits

Several factors could have contributed the above result, such as the proximity of the cut site to the edit site as well as the presence of silent mutations in the ssODN to prevent re-editing after successful editing. Further, because the plasmid-based method relies on proper generation of both the gRNA and Cas9 gene in addition to the formation of the Cas9:gRNA complex in the cell, editing efficiencies can be limited [21]. To increase editing efficiencies, we employed a pre-formed ribonucleoprotein (RNP) complex approach that consists of Cas9 protein and the gRNA. The RNP complex, once transfected, is ready to begin editing upon entry into the nucleus, thus increasing its efficiency. To enhance editing efficiencies, we also used the second gRNA design (#2 gRNA, Table S1) that reduces distance between the cut and the edit site (Fig. 2A). In addition, a silent mutation within the gRNA sequence was included in the ssODN DONOR template to prevent re-editing (Fig. 2A, S2) [22]. Among the 78 clones sequenced, the majority of mutations using this method were “mixed pool” mutations, however, insertion mutations were greatly reduced. Importantly, editing did result in heterozygous mutations containing both the desired knock-in and the silent mutations (Fig. 2C, D, Table S3 and S4). The majority of indel mutations occurred surrounding the Cas9 cut site, the silent mutation site, or the desired edit site (Fig. 2B).

Although this method failed to produce homozygous mutants in a single round (RNP 1), the presence of the silent mutation enabled us to continue to generate homozygous mutants from the heterozygous mutation cell line [22]. Indeed, when the heterozygous T55A cell line was re-transfected with the same components above in the second round (RNP 2), the homozygous mutation of the desired edit, together with silent mutation, was obtained (Fig. 2C, D). As expected, among 68 clones sequenced, the majority of indel mutations flank the Cas9 cut site, which is 10 bp from the target edit (Fig. 2B). Those studies suggested that RNP-based approach is more effective in generating single-base pair knock-in mutations.

### Adenosine Base editors (ABE) fail to produce genomic editing

Next, we assess a new technology using adenosine base-editors for specific editing. A tRNA adenine deaminase (TadA) fused to a catalytically impaired *sp*Cas9 nuclease forms the spCas9-ABE7.9 base editor capable of making single base pair edits. Because this method does not rely on CRISPR/Cas9 mediated DSBs, indel mutations are largely eliminated. The specific ABEs can successfully convert adenine to guanine (A to G), by first converting A to inosine (I), which is later converted to G through DNA repair or replication (Figure S3) [15]. The spCas9-ABE7.9 forms a complex with the gRNA, which is designed to target and edit A bases located between bases 4–7 of the gRNA, where the PAM sequence spans bases 21–23 (Figure S3A). Efficiency of the base editing system increases based on the location of the desired edit, where base 4–7 within the gRNA gives the best chance for editing and bases 8–10 are less efficient. Due to the location of our target site and available gRNAs, we were limited in the placement of our target site at base 10 of the gRNA (Fig. S3A). Perhaps due to the proximity limitation of the desired edit site in respects to the TadA domain of the base editor no editing was observed upon screening of 100 clones (Fig. 4A). To overcome this issue, the xCas9-ABE7.10 adenosine base editor was utilized (Figure S3B) [23]. The xCas9 nuclease has been evolved to broaden the PAM sequence capabilities so that it can recognize “NGN” rather than the classic “NGG”, allowing our desired target to fall within the base 4 location (Fig. S3B). However, in spite of ideal editing conditions, no editing was obtained using this system upon screening of 66 clones (Fig. 4A).

### Editing using a neomycin cassette coupled homologous recombination results in highly specific heterozygous mutations

**Fig. 3:**
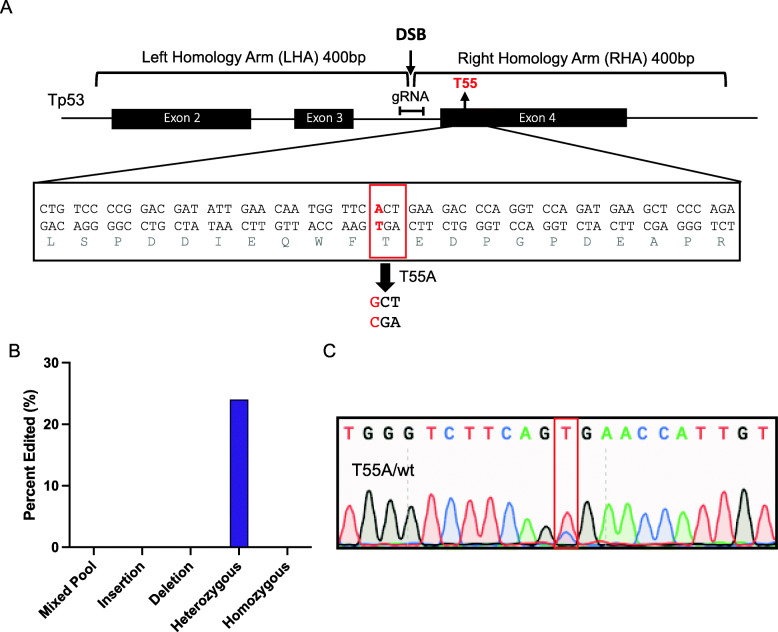
Editing using a neomycin cassette coupled homologous recommendation results in highly specific heterozygous mutations. **A** Schematic presentation of left homology arm (LHA), right homology arm (RHA), and gRNA in respect to location of T55 residue (red box). **B**, **C** Percentage of specific mutations and chromatogram confirming heterozygous T55A mutation. Red box indicates single base pair target. Note data represents sequence data of template strand

In an effort to increase the likelihood of obtaining our edit, we explored a knock-in system that would allow for selection of clones that had specifically been edited. Cells were transfected with the same co-expression plasmid as our traditional method, in addition to a DONOR plasmid. The DONOR plasmid contains a neomycin cassette flanked by loxP sites, and two 400 bp homology arms, one of which (right homology arm, RHA) contains the target edit (Fig. S4). Upon CRISPR/Cas9 mediated DSBs, the neomycin cassette and the edit are incorporated into the genome through homologous recombination, thus decreasing the likelihood of indel mutations. To avoid disruption of the p53 gene, the cassette was inserted into intron 3 using a gRNA within 100 bp of the desired edit site to ensure editing (Fig. 3A). Furthermore, to ensure proper splicing, the neomycin cassette was floxed and removed upon Cre-mediated recombination (Fig. S4). Utilizing this neomycin knock-in method, DNA was isolated from 125 clones and sequenced. The analysis shows that 24% of the clones contain desired mutations, and importantly, no indel mutations were observed within the area sequenced (Fig. 3B). However, although there was increased specificity to editing of the target site, all of the edits only resulted in heterozygous mutations (Fig. 3B, C, Table S3 and S4). This result suggests inefficiencies in the traditional plasmid-based method to target both copies of DNA. This approach, however, provides an efficient tool for generating highly specific heterozygous mutations.

## Discussion

In this study, we evaluated several commonly used CRISPR/Cas9 techniques for their effectiveness on making a single base pair knock-in edit. Traditional plasmid-based methods where a plasmid co-expresses the gRNA and Cas9 protein results in mutations largely caused by the NHEJ repair pathway. Even in the presence of a NHEJ inhibitor, “mixed pool”, insertion and deletions, surrounding the Cas9 cut site dominated the resulting edits. The data suggest that, among all the methods evaluated in our study, the plasmid-based approach is most efficient in knocking out genes.

While adenosine base-editors have the advantage in simplicity, given that they do not require a DONOR template, the lack of editing implies it is a less efficient method. Low editing efficiencies of the original spCas9/ABE7.9 were most likely due to the fact our edit site could not be designed to fall within the range in the gRNA that was needed for maximum editing. However, the xCas9/ABE7.10 protein has a broader range of PAM sequences (NGN) that allowed us to have our edit within the necessary range, but lack of edits suggests it is less efficient in base editing in our study. Of note, while xCas9 was originally reported to target NGN PAMs, it has recently been suggested to have PAM preference of NGGC [24]. This could contribute to low editing in our study. Other NGN variants, such as SpCas9-NG [25] and SpG [24] have also been reported to target NGN PAMs. Clearly, it will be intriguing to assess those variants to target our NGAT target site in the future.

To increase the likelihood of obtaining the desired knock-in edits, we explored an approach to facilitate selection of clones containing editing. This approach can be used for single base pair edits or be modified for any knock-in edit (i.e., multiple base pairs or protein tags). While this method helps to select for clones that have undergone editing, it requires the target edit site to be within 100 bp of an intron. In addition, integration of large repair templates can be inefficient for editing both alleles of a cell simultaneously. Despite its limitation, however, our results show that this method results in highly specific heterozygous mutations, and no indel mutations were found. Combination of pre-formed RNP could potentially increase editing efficiency and make this method feasible for base-specific editing, particularly for generating highly specific heterozygous mutations.

## Conclusion

**Fig. 4:**
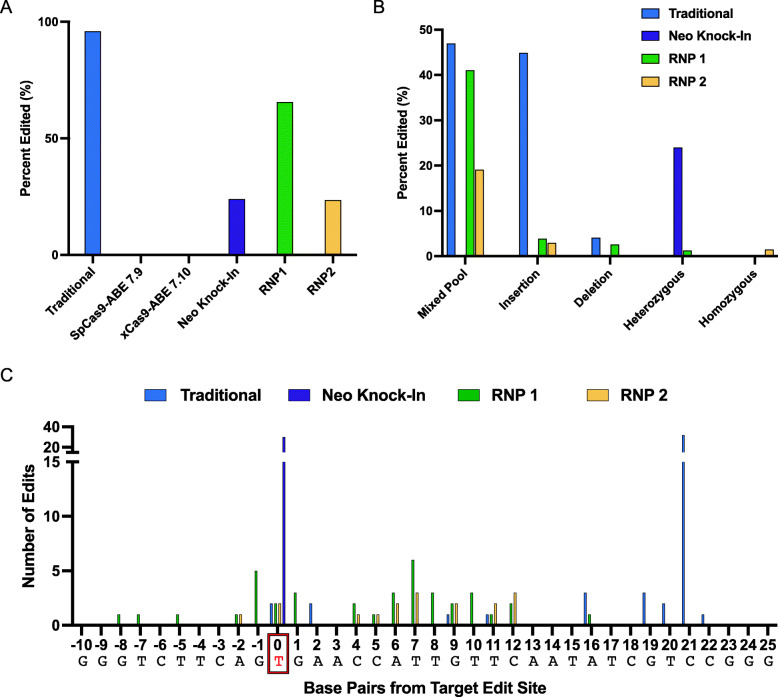
Analysis of mutation pattern among different CRISPR/Cas9 genome editings. **A** Comparison of overall edits made by varying methods of CRISPR/Cas9 editing. **B**, **C** Comparison of specific mutations (**B**) and location of edits (**C**) using different CRISPR/Cas9 editing methods

Analysis of several CRISPR/Cas9-mediated genome editing methods revealed that, although traditional plasmid-based method has the highest percent editing (Fig. 4A), the high occurrence of “mixed pool”, insertion and deletion mutations (Fig. 4B) and lack of specific target edits (Fig. 4C) lead it to be a less desirable method in generating a single-base pair knock-in mutation on tumor suppressor p53. To improve this method, we found that use of the RNP complex, a gRNA that reduces the distance between the cut and the edit site, and inclusion of a silent mutation increased efficiency of the single base pair edit. Adenosine base editors resulted in a complete lack of editing (Fig. 4A), suggesting that the limitations in our design with the necessary requirements for this method are not likely compatible. Although our knock-in neomycin cassette method only resulted in heterozygous mutations (Fig. 4B), this method circumvents the problem of indel mutations, and allows for the highest specificity to the target location. The most efficient method found in our study in generating single-base pair knock-in mutations was RNP, which resulted in increased specificity for target edit sites (Fig. 4C) and yielded the desired homozygous mutant (Fig. 4B). Given that generating single-base pair knock-in mutations is critical in understanding function of posttranslational modification, our provided additional guidance for those studies.

## Methods

### Traditional co-expression method

Prospective gRNAs were designed using the Zhang lab CRISPR Design tool (crispr.mit.edu). Following the selection, the top choice was cloned into pSpCas9(BB)-2A_Puro plasmid (Addgene, #62988). Single stranded oligonucleotide (ssODN) (100 bp) was designed to have 50 bp flanking the DSBs (Ultramer DNA Oligo; IDT). HCT-116 cells were seeded into 24-well plates to be 70% 12-14 h following seeding. Co-transfection of CRISPR plasmid (2μg), and ssODN template (4ul of 10 mM stock) was performed using Lipofectamine 3000 in the presence of non-homologous end joining (NHEJ) SCR7 inhibitor (0.2uM, HY-12742 Med Chem Express). Following transfection, cells were selected with puromycin (1μg/ml) for 24 h before clonal expansion (see below).

### Ribonucleoprotein (RNP) method

For the RNP method we utilized the Alt-R CRISPR-Cas9 technology from IDTDNA. gRNA used in RNP method was a verified guide (Alt- R CRISPR- Cas9 crRNA, 2 nmol; Hs.Cas9.TP53.1.AK). RNP complex included crRNA; tracrRNA; and Cas9 nuclease (Alt-R CRISPR-Cas9 tracrRNA, #1072532; Alt-R S.p. Cas9 Nuclease V3, #1081058). Single stranded oligonucleotide (ssODN) DONOR template was designed to include T55A and silent mutation. Total length was 78 bp with arms flanking the DSB so that mutation sites were centralized (IDTDNA). HCT-116 cells were seeded into a 12-well plate to be 70% confluent 12-14 h following seeding. The RNP complex was preformed according to the Alt-R CRISPR-Cas9 protocol. RNP complex and DONOR ssODN (1uM) were transfected using RNAiMAX (Thermo #13778100) in the presence of 20uM Alt-R HDR Enhancer (IDTDNA # 1081072).

### Adenosine base editor (ABE) method

gRNA was designed manually following the parameters from [15, 23], and cloned into BPK1520 sgRNA expression plasmid (Addgene, #65777). HCT-116 cells were seeded on 48-well plates to be 70% confluent 12-14 h following seeding. Co-transfection of ABE plasmid (750 ng), gRNA expression plasmid (250 ng) was performed using Lipofectamine 3000. pCMV-ABE7.9 and xCas-(3.7)-ABE (7.10) plasmids were from Addgene (#102918; #108382).

### Neomycin knock-in method

gRNA was designed manually in intron 3 of the Tp53 gene and cloned into the pSpCas9(BB)-2A_Puro (PX459) plasmid. For DONOR plasmid, 400 bp homology arms flanking the DSB were cloned into pGolden-Neo plasmid, which contains the floxed neomycin cassette. Site directed mutagenesis was used to create T55A mutation in the right homology arm (RHA). HCT-116 cells were seeded into a 6-well plate to be 70% confluent 12-14 h following seeding. Co-transfection of spCas9 (1μg) and DONOR plasmid (1μg) was transfected using Lipofectamine 3000. Following transfection, cells were selected for 72 h using 1μg/ml neomycin before clonal expansion (see below). After confirmation of heterozygous mutations through Sanger Sequencing, clones were transfected with pCMV-CRE plasmid to remove Neomycin cassette.

### Clonal expansion, DNA extraction, and sanger sequencing

Selected clonal cells were diluted to have a final concertation of 1 cell/well, seeded into 96-well plates, and allowed to grow for 2 weeks, or until the appearance of rounded colonies. Single colonies were harvested using trypsin and DNA was extracted using QuickExtract (Lucigen #QE09050) buffer. Extracted DNA was then prepped for sequencing. PCR amplicons containing sequence flanking Thr55 were purified using DNA clean & concentrator (Zymo #D4013) and Sanger sequenced. Primer set used to amplify sequence flanking Thr55 are:

- FOR 5′ – GCAGTCAGATCCTAGCGTCG
- REV 5′ - TACGGCCAGGCATTGAAGT

## Supplementary Information

**Additional file 1: Supplemental Figure 1.** Schematic of traditional plasmid-based method of CRISPR/Cas9 genome editing for T55A. **Supplemental Figure 2.** Design of Ribonucleoprotein (RNP) complex for targeting T55. **Supplemental Figure 3.** Design of Adenosine Base Editors (ABE) for targeting T55. **Supplemental Figure 4.** Base editing using a selectable neomycin cassette coupled homologous recombination. **Supplemental Table 1.** Top choices of gRNA. **Supplemental Table 2.** gRNA and DONOR sequences. **Supplemental Table 3.** Experimental replicate data. **Supplemental Table 4.** Summary of sequencing results of each colonies.

## Data Availability

The sequence data used during the current study are available from the corresponding authors upon reasonable request.

## References

[1] Pawson T, Scott JD (2005). Protein phosphorylation in signaling – 50 years and counting. Trends Biochem Sci.

[2] Nair A, Chauhan P, Saha B, Kubatzky KF. Conceptual evolution of cell signaling. Int J Mol Sci. 2019;20(13) 4 [cited 2020 Aug 8]. Available from: https://www.ncbi.nlm.nih.gov/pmc/articles/PMC6651758/. 10.3390/ijms20133292 PMC6651758 31277491

[3] Tarrant MK, Cole PA (2009). The chemical biology of protein phosphorylation. Annu Rev Biochem.

[4] Christian M, Cermak T, Doyle EL, Schmidt C, Zhang F, Hummel A, Bogdanove AJ, Voytas DF (2010). Targeting DNA double-Strand breaks with TAL effector nucleases. Genetics..

[5] Flotte TR (2004). Gene therapy Progress and prospects: recombinant adeno-associated virus (rAAV) vectors. Gene Ther.

[6] Miller JC, Holmes MC, Wang J, Guschin DY, Lee Y-L, Rupniewski I, Beausejour CM, Waite AJ, Wang NS, Kim KA, Gregory PD, Pabo CO, Rebar EJ (2007). An improved zinc-finger nuclease architecture for highly specific genome editing. Nat Biotechnol.

[7] Porteus MH, Baltimore D (2003). Chimeric nucleases stimulate gene targeting in human cells. Science..

[8] Stoddard BL (2006). Homing endonuclease structure and function. Q Rev Biophys.

[9] Horvath P, Barrangou R (2010). CRISPR/Cas, the immune system of bacteria and archaea. Science..

[10] Makarova KS, Wolf YI, Iranzo J, Shmakov SA, Alkhnbashi OS, Brouns SJJ, Charpentier E, Cheng D, Haft DH, Horvath P, Moineau S, Mojica FJM, Scott D, Shah SA, Siksnys V, Terns MP, Venclovas Č, White MF, Yakunin AF, Yan W, Zhang F, Garrett RA, Backofen R, van der Oost J, Barrangou R, Koonin EV (2020). Evolutionary classification of CRISPR–Cas systems: a burst of class 2 and derived variants. Nat Rev Microbiol.

[11] Cong L, Ran FA, Cox D, Lin S, Barretto R, Habib N, Hsu PD, Wu X, Jiang W, Marraffini LA, Zhang F (2013). Multiplex genome engineering using CRISPR/Cas systems. Science..

[12] Hsu PD, Scott DA, Weinstein JA, Ran FA, Konermann S, Agarwala V, Li Y, Fine EJ, Wu X, Shalem O, Cradick TJ, Marraffini LA, Bao G, Zhang F (2013). DNA targeting specificity of RNA-guided Cas9 nucleases. Nat Biotechnol.

[13] Chang HHY, Pannunzio NR, Adachi N, Lieber MR (2017). Non-homologous DNA end joining and alternative pathways to double-strand break repair. Nat Rev Mol Cell Biol.

[14] Jasin M, Rothstein R. Repair of strand breaks by homologous recombination. Cold Spring Harb Perspect Biol. 2013;5(11) [cited 2020 Aug 8]. Available from: https://www.ncbi.nlm.nih.gov/pmc/articles/PMC3809576/. 10.1101/cshperspect.a012740 PMC3809576 24097900

[15] Gaudelli NM, Komor AC, Rees HA, Packer MS, Badran AH, Bryson DI, Liu DR (2017). Programmable base editing of a•T to G•C in genomic DNA without DNA cleavage. Nature..

[16] Bode AM, Dong Z (2004). Post-translational modification of p53 in tumorigenesis. Nat Rev Cancer.

[17] Li H-H, Li AG, Sheppard HM, Liu X (2004). Phosphorylation on Thr-55 by TAF1 mediates degradation of p53: a role for TAF1 in cell G1 progression. Mol Cell.

[18] Wu Y, Lin JC, Piluso LG, Dhahbi JM, Bobadilla S, Spindler SR, Liu X (2014). Phosphorylation of p53 by TAF1 inactivates p53-dependent transcription in the DNA damage response. Mol Cell.

[19] Hu Z, Shi Z, Guo X, Jiang B, Wang G, Luo D, et al. Ligase IV inhibitor SCR7 enhances gene editing directed by CRISPR–Cas9 and ssODN in human cancer cells. Cell Biosci. 2018;8(1). 10.1186/s13578-018-0200-z PMC5819182 29468011

[20] Brinkman EK, Chen T, Amendola M, van Steensel B (2014). Easy quantitative assessment of genome editing by sequence trace decomposition. Nucleic Acids Res.

[21] Kim S, Kim D, Cho SW, Kim J, Kim J-S (2014). Highly efficient RNA-guided genome editing in human cells via delivery of purified Cas9 ribonucleoproteins. Genome Res.

[22] Paquet D, Kwart D, Chen A, Sproul A, Jacob S, Teo S, Olsen KM, Gregg A, Noggle S, Tessier-Lavigne M (2016). Efficient introduction of specific homozygous and heterozygous mutations using CRISPR/Cas9. Nature..

[23] Hu JH, Miller SM, Geurts MH, Tang W, Chen L, Sun N, Zeina CM, Gao X, Rees HA, Lin Z, Liu DR (2018). Evolved Cas9 variants with broad PAM compatibility and high DNA specificity. Nature..

[24] Walton RT, Christie KA, Whittaker MN, Kleinstiver BP (2020). Unconstrained genome targeting with near-PAMless engineered CRISPR-Cas9 variants. Science..

[25] Nishimasu H, Shi X, Ishiguro S, Gao L, Hirano S, Okazaki S, Noda T, Abudayyeh OO, Gootenberg JS, Mori H, Oura S, Holmes B, Tanaka M, Seki M, Hirano H, Aburatani H, Ishitani R, Ikawa M, Yachie N, Zhang F, Nureki O (2018). Engineered CRISPR-Cas9 nuclease with expanded targeting space. Science..

