# Supplementary material for: Analysis of conventional and alternative CRISPR/Cas9 genome editing to enhance a single-base pair knock-in mutation
Source: BMC Biotechnol. 2021 Jul 27;21:45. doi: 10.1186/s12896-021-00707-5 (PMC8317408; doi:10.1186/s12896-021-00707-5)
Supplement: Supplementary file 1 — Additional file 1: Supplemental Figure 1. Schematic of traditional plasmid-based method of CRISPR/Cas9 genome editing for T55A. Supplemental Figure 2. Design of Ribonucleoprotein (RNP) complex for targeting T55. Supplemental Figure 3. Design of Adenosine Base Editors (ABE) for targeting T55. Supplemental Figure 4. Base editing using a selectable neomycin cassette coupled homologous recombination. Supplemental Table 1. Top choices of gRNA. Supplemental Table 2. gRNA and DONOR sequences. Supplemental Table 3. Experimental replicate data. Supplemental Table 4. Summary of sequencing results of each colonies. [file 12896_2021_707_MOESM1_ESM.pdf]

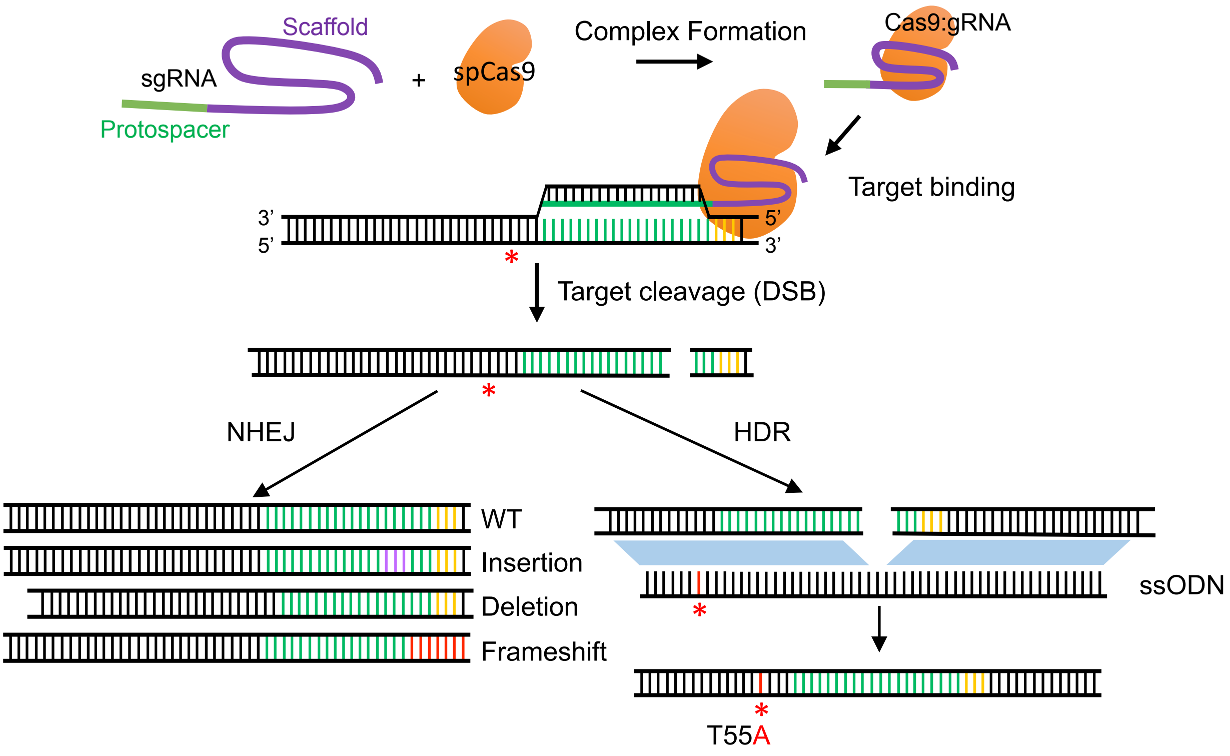

Supplemental Figure 1. Schematic of traditional plasmid-based method of CRISPR/Cas9 genome editing for T55A. The gRNA (green) forms a complex with the Cas9 nuclease to bind target genomic DNA. Upon target cleavage, double stranded breaks (DSB) can be repaired by error-prone Non-Homologous End Joining (NHEJ) or error-free Homology Directed Repair (HDR). NHEJ DNA repair results in mutations including insertion, deletions, and frameshifts, while HDR results with the desired edit. HDR utilizes a 100bp single stranded oligonucleotide (ssODN) which harbors the T55A mutation.

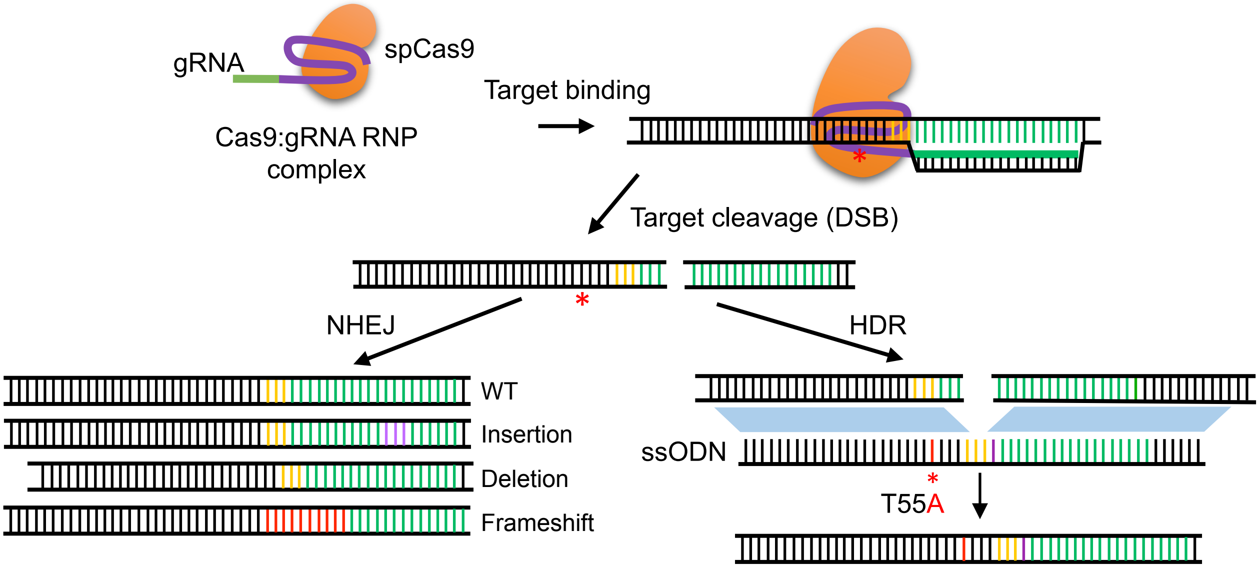

Supplemental Figure 2. Design of Ribonucleoprotein (RNP) complex for targeting T55. Upon target and cleavage by RNP, double stranded breaks (DSB) can be repaired by error-prone Non-Homologous End Joining (NHEJ) or Homology directed repair (HDR). HDR utilizes a single stranded oligonucleotide (ssODN) that harbors the T55A mutation and a silent mutation {brown) in the gRNA sequence {green).

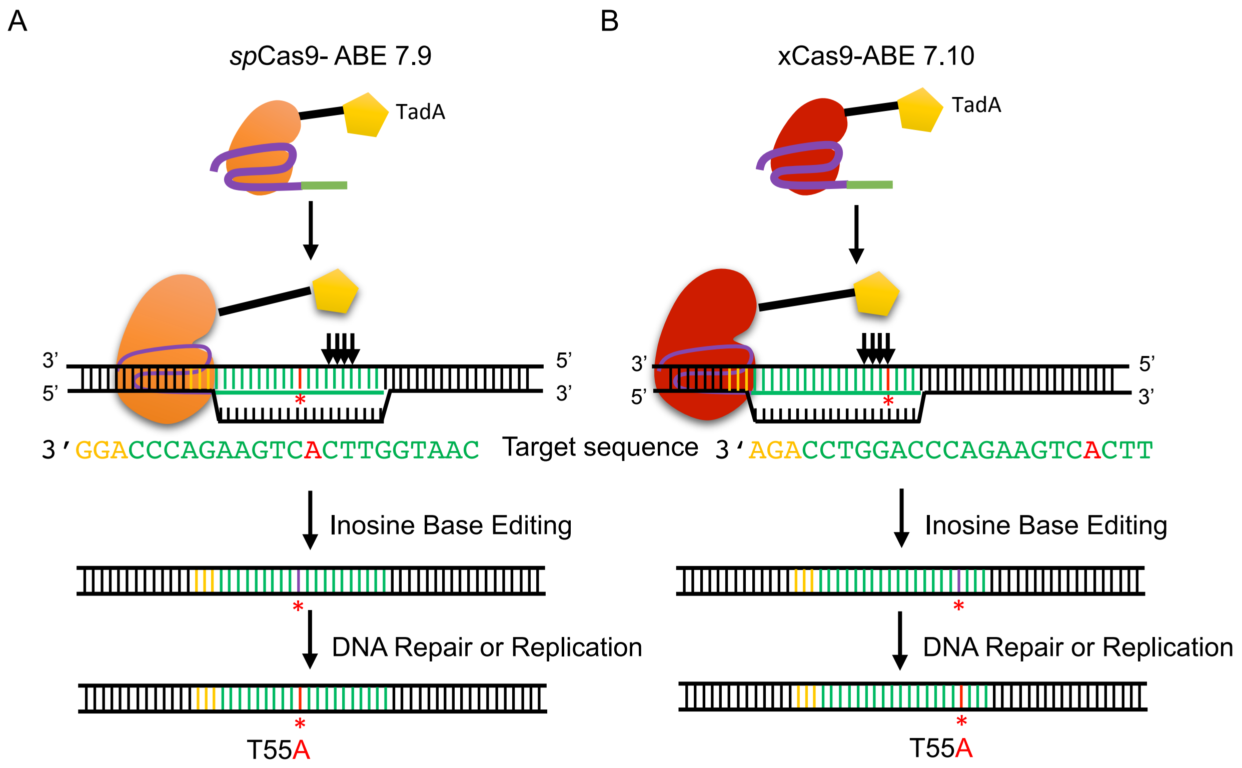

Supplemental Figure 3. Design of Adenosine Base Editors (ABE) for targeting T55. (A) Schematic of A to G base editing using ABE7.9. A tRNA adenine deaminase (TadA) domain is fused with catalytically dead *sp*Cas9 (dCas9) nuclease to form the *sp*Cas9- ABE7.9 which forms a complex with the gRNA (green) to target genomic DNA. (B) Schematic of A to G base editing using ABE7.10. A TadA domain is fused to catalytically dead *sp*Cas9 variant with broad PAM capabilities (xCas9) to form xCas9-ABE 7.10. Green and orange denote gRNA and PAM (AGA) in respect to location of T55 (red *). Black arrows denote desired editing positions.

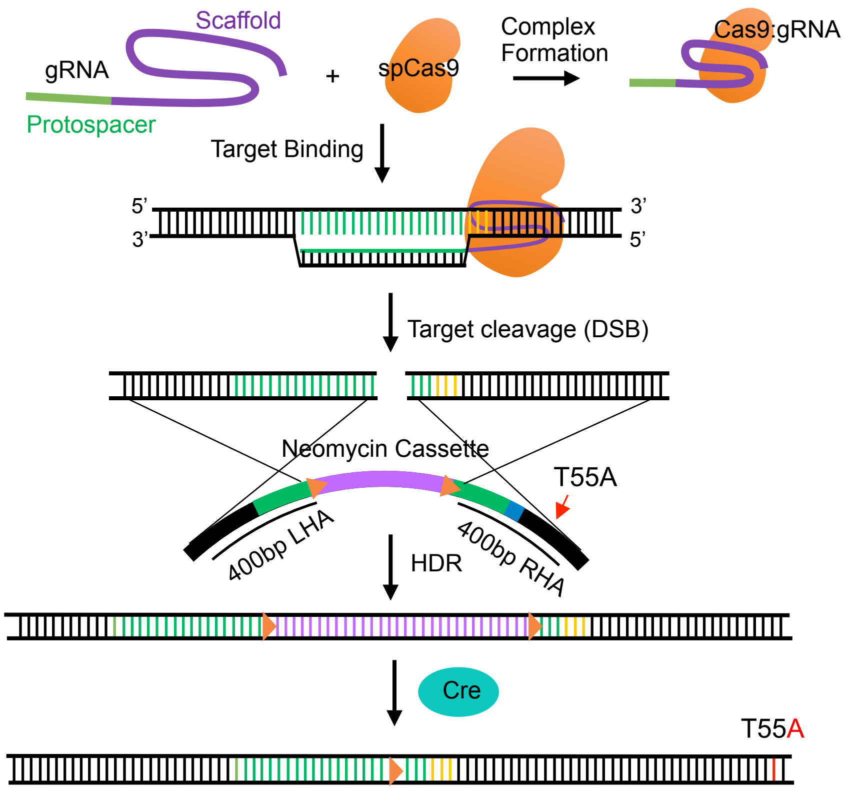

Supplemental Figure 4. Base editing using a selectable neomycin cassette coupled homologous recombination. Cas9:gRNA complex binds target genomic DNA and cleaves DNA. The resulting double stranded break (DSB) is repaired through Homology Directed Repair (HDR) using a donor template DNA containing a left homology arm (LHA), a right homology arm (RHA) carrying the T55A mutation, and a neomycin cassette flanked by loxP sites. After selection, Cre recombinase is introduced to remove neomycin cassette.

	Potential Guide	Quality Score
gRNA #1	5′ CCATTGTTCAATATCGTCCG	97
gRNA #2	5′ CCCCGGACGATATTGAACAA	91
gRNA #3	5′ GGCATTCTGGGAGCTTCATC	70
gRNA #4	5′ CAATGGTTCACTGAAGACCC	67

Supplemental Table 1. Top choices of gRNA.

Method	gRNA	Sequences used for DONOR template
Traditional	5′ CCATTGTTCAATATCGTCCG	ssODN:<br>5′GGGAGCTTCATCTGGACCTGGGTCTTCAG**C**GAACCATTGT<br>TCAATATCGTCCGGGGACAGCATCAAATCATCCATTGCTTGG<br>GACGGCAAGGGGGA
RNP	5′ CCCCGGACGATATTGAACAA	ssODN:<br>5′GATGATTTGATGCTGTCCCCGGACGATATTGAACA**G**TGGT<br>TC**G**CTGAAGACCCAGGTCCAGATGAAGCTCCCAGAATG
spCas9-ABE7.9	5′ CAATGGTTCACTGAAGACCC	N/A
xCas9-ABE7.10	5′ TTCACTGAAGACCCAGGTCC	N/A
Neo Knock-In	5′ TGGGTGAAAAGAGCAGTCAG	LHA:<br>GTTCCTTCTCTGCAGGCCCAGGTGACCCAGGGTTGGAAG<br>TGTCTCATGCTGGATCCCCACTTTTCCTCTTGCAGCAGCCAG<br>ACTGCCTTCCGGGTCACTGCCATGGAGGAGCCGCAGTCAGAT<br>CCTAGCGTCGAGCCCCCTCTGAGTCAGGAAACATTTTCAGAC<br>CTATGGAAACTGTGAGTGGATCCATTGGAAGGGCAGGCCCAC<br>CACCCCCACCCCAACCCCAGCCCCCTAGCAGAGACCTGTGGG<br>AAGCGAAAATTCCATGGGACTGACTTTCTGCTCTTGTCTTTC<br>AGACTTCCTGAAAACAACGTTCTGGTAAGGACAAGGGTTGGG<br>CTGGGGACCTGGAGGGCTGGGGACCTGGAGGGCTGGGGGGCT<br>GGGGGGCTGAGGACCTGGTCCTCTG<br>RHA:<br>ACTGCTCTTTTCACCCATCTACAGTCCCCCTTGCCGTCCCAA<br>GCAATGGATGATTTGATGCTGTCCCCGGACGATATTGAACAA<br>TGGTTC**G**CTGAAGACCCAGGTCCAGATGAAGCTCCCAGAATG<br>CCAGAGGCTGCTCCCCCCGTGGCCCCTGCACCAGCAGCTCCT<br>ACACCGGCGGCCCCTGCACCAGCCCCCTCCTGGCCCCTGTCA<br>TCTTCTGTCCCTTCCCAGAAAACCTACCAGGGCAGCTACGGT<br>TTCCGTCTGGGCTTCTTGCATTCTGGGACAGCCAAGTCTGTG<br>ACTTGCACGGTCAGTTGCCCTGAGGGGCTGGCTTCCATGAGA<br>CTTCAATGCCTGGCCGTATCCCCCTGCATTTCTTTTGTTTGG<br>AACTTTGGGATTCCTCTTCACC

Supplemental Table 2. gRNA and DONOR sequences

	Traditional					RNP 1				RNP 2		Neo KI	
	Rep 1	Rep 2	Rep 3	Sum	%	Rep 1	Rep 2	Sum	%	Rep 1	%	Rep 1	%
Mixed Pool	4	10	9	23	**46.94**	18	14	32	**41.03**	13	**19.12**	0	**0**
Insertion	14	3	5	22	**44.90**	1	2	3	**3.85**	2	**2.94**	0	**0**
Deletion	0	1	1	2	**4.08**	0	2	2	**2.56**	0	**0.00**	0	**0**
T55A Hetero	0	0	0	0	**0.00**	0	1	1	**1.28**	0	**0.00**	30	**24**
T55A Homo	0	0	0	0	**0.00**	0	0	0	**0.00**	1	**1.47**	0	**0**
WT	0	2	0	2	**4.08**	10	30	40	**51.28**	52	**76.47**	95	**76**
Total Clones	18	16	15	49	**N/A**	29	49	78	**N/A**	68	**N/A**	125	**N/A**
Total Edited	18	14	15	47	**95.92**	19	19	38	**65.52**	16	**23.53**	30	**24**

Supplemental Table 3. Experimental replicate data. Traditional: traditional plasmid-based method; RNP: Ribonucleoprotein complex; Neo KI: Neomycin cassette coupled homologous recombination; Rep: replicates.

	Traditional				RNP 1			RNP 2	Neo KI
Edit Site	Rep 1	Rep 2	Rep 3	Sum	Rep 1	Rep 2	Sum	Rep 1	Rep 1
-8	0	0	0	0	0	1	1	0	0
-7	0	0	0	0	1	0	1	0	0
-6	0	0	0	0	0	0	0	0	0
-5	0	0	0	0	1	0	1	0	0
-4	0	0	0	0	0	0	0	0	0
-3	0	0	0	0	0	0	0	0	0
-2	0	0	0	0	1	0	1	1	0
-1	0	0	0	0	2	3	5	0	0
0	0	2	0	2	1	1	2	2	30
1	0	0	0	0	1	2	3	0	0
2	0	0	2	2	0	0	0	0	0
3	0	0	0	0	0	0	0	0	0
4	0	0	0	0	1	1	2	1	0
5	0	0	0	0	0	1	1	1	0
6	0	0	0	0	1	2	3	2	0
7	0	0	0	0	2	4	6	3	0
8	0	0	0	0	3	0	3	0	0
9	0	1	0	1	2	0	2	2	0
10	0	0	0	0	3	0	3	0	0
11	0	1	0	1	0	1	1	2	0
12	0	0	0	0	2	0	2	2	0
13	0	0	0	0	0	0	0	0	0
14	0	0	0	0	0	0	0	0	0
15	0	0	0	0	0	0	0	0	0
16	0	0	3	3	0	1	1	0	0
17	0	0	0	0	0	0	0	0	0
18	0	0	0	0	0	0	0	0	0
19	0	0	3	3	0	0	0	0	0
20	0	1	1	2	0	0	0	0	0
21	17	9	6	32	0	0	0	0	0
22	1	0	0	1	0	0	0	0	0

Supplemental Table 4. Summary of sequencing results of each colonies. Traditional: traditional plasmid-based method; RNP: Ribonucleoprotein complex; Neo KI: Neomycin cassette coupled homologous recombination; Edit site: “0” denotes designated target site; Rep: replicates.
